# Kratom-Induced Cholestatic Liver Injury and Its Conservative Management

**DOI:** 10.1177/2324709619836138

**Published:** 2019-03-28

**Authors:** Christopher T. Fernandes, Umair Iqbal, Sean P. Tighe, Aijaz Ahmed

**Affiliations:** 1 Stanford University, Stanford, CA, USA; 2 Geisinger Commonwealth School of Medicine, Scranton, PA, USA

**Keywords:** kratom, drug-induced liver injury, hyperbilirubinemia, herbal supplements

## Abstract

Drug-induced liver injury (DILI) is a common cause of hepatotoxicity associated with prescription-based and over-the-counter exposure to medications and herbal supplements. Use of unapproved and inadequately tested herbal supplements can cause DILI. Therefore, thorough history-taking on exposure to herbal supplements must be an integral part of clinical evaluation of DILI. Kratom is an herbal supplement or remedy that has been known for its analgesic effects and has also been used for self-treatment of opiate withdrawals. A 52-year-old man was seen for evaluation of yellow discoloration of the eyes and skin. He reported taking kratom for right shoulder strain for at least a couple of months. On workup, his total bilirubin was noted to be 23.2 mg/dL, which peaked at 28.9 mg/dL. He was noted to have mild elevation of aspartate aminotransferase, alanine aminotransferase, and alkaline phosphatase. Extensive laboratory tests were ordered and known causes of chronic liver disease ruled out. Magnetic resonance imaging of the abdomen was unremarkable without stigmata of portal hypertension or signs of chronic liver disease. He demonstrated no evidence of coagulopathy or hepatic encephalopathy during his illness. He underwent liver biopsy, which demonstrated histologic evidence of acute cholestatic hepatitis highly suspicious of DILI. He was advised to avoid kratom or other herbal supplements in future and prescribed ursodeoxycholic acid with significant improvement in his liver chemistries. Kratom is associated with significant liver enzymes derangements leading to DILI. Kratom is not approved for use in the United States and should be avoided.

## Introduction

Drug-induced liver injury (DILI) is one of the most common cause of acute liver failure in the United States.^1,2^ Variety of prescription and nonprescription drugs are associated with DILI. Herbal and dietary supplements are a frequent cause of DILI.^3^ Majority of these herbal supplements are marketed as dietary products and not regulated by the Food and Drug Administration (FDA; a federal agency of the US Department of Health and Human Services). Due to lack of strict manufacturing regulations and oversight of the herbal supplements, dosing variation in formulations of herbal products is inevitable and may result in inconsistencies in the final chemical composition of a herbal supplement and other ingredients or preservatives added to it. This may increase the risk of adverse events and toxicities associated with herbal products. Individuals can self-medicate themselves with these seemingly harmless herbal supplement products due to attractive promotions, easy access, lack of knowledge of potential adverse effects, and without consulting their physicians. It is not uncommon for patients to forget reporting the use of herbal supplements when they present with hepatic dysfunction. Therefore, it is important to obtain a meticulous history of consumption of dietary supplements during the clinical evaluation of patients with hepatic dysfunction and suspicion of DILI. Kratom is a herbal supplement that comes from the plant *Mitragyna speciosa* and is known for its therapeutic role as an analgesic with opiate-like properties.^4^ It has also been used for self-treatment of symptoms of opioid withdrawal.^4^ Given the lack of standardized clinical trials assessing the safety, tolerability, dosing regimen, adverse effects profile, and toxicity associated with kratom, it should be avoided until further data are generated. We report here a case of idiosyncratic, acute cholestatic hepatic injury secondary to use of kratom.

## Case Report

A 52-year-old man presented for clinical evaluation of progressive yellow discoloration of eyes and skin over the course of 2 months. He was in his usual state of health until August 2018, when he strained his right shoulder. For symptomatic pain relief, he started taking kratom, an over-the-counter herbal supplement for pain control. He reported taking kratom, along with acetaminophen, for almost 2 months as 800 mg of acetaminophen twice daily alone did not alleviate his pain. He started taking kratom, at first twice a day for a few days, and then once a day. He took kratom in the form of crushed leaves with water, 1 teaspoonful (approximately 1.5 g) daily. He was on kratom from early August until October 6, 2018, for about 2 months. At presentation on October 22, 2018, he reported mild fatigue. According to his friends, there were no changes in mental status suspicious for hepatic encephalopathy. He was found to have an elevated bilirubin level of 23.2 mg/dL on workup of his jaundice. Total bilirubin peaked approximately 10 days later at 28.9 mg/dL. International normalizes ratio levels were within normal range. Aspartate aminotransferase was 55 U/L, alanine aminotransferase 66 U/L, alkaline phosphatase 255 U/L, and lipase 156 U/L. Magnetic resonance imaging of the abdomen did not reveal any signs of intrinsic liver disease and showed patent biliary ducts. Workup for known causes of chronic liver disease was negative. A liver biopsy was performed, which showed an adequate number of portal tracts present for evaluation. There was marked canalicular cholestasis. The portal tracts contained mixed inflammation of lymphocytes, eosinophils, and some neutrophils. Some bile ducts showed features of injury, such as epithelial disarray, cytoplasmic eosinophilia, and lymphocytes within the epithelium. There was mild bile ductular reaction without loss of bile ducts. No appreciable interface activity or portal edema was identified. The hepatic lobules possessed features of recent injury, including a mild sinusoidal mononuclear infiltrate, Kupffer cell hyperplasia, and lobular and periportal ceroid macrophages. Rare acidophil bodies and rare foci of spotty necrosis were noted. No significant steatosis was appreciated. Trichrome stain revealed no histological evidence of fibrosis. Diastase-pretreated periodic acid-Schiff stain highlighted ceroid macrophages but did not demonstrate periportal eosinophilic globules, lending no support for α-1-antitrypsin deficiency. Rhodanine stain was negative for copper deposition. Prussian blue stain showed 2+ stainable hepatocellular and Kupffer cell iron. Reticulin stain revealed preserved hepatic plate thickness. Overall, it was suspected that these findings were consistent with acute cholestatic injury as a result of kratom-related DILI. He was prescribed 600 mg of ursodeoxycholic acid (UDCA), 3 times daily and followed conservatively. He was advised to avoid kratom and any other herbal supplemental products in future.

At a 2-week follow-up visit in the hepatology clinic, he denied any signs and symptoms of clinical deterioration. His energy level improved, and mental status remained intact. His physical examination was unremarkable except for deeply icteric sclerae. His follow-up bilirubin was higher and peaked at 28.9 mg/dL. He was followed closely with biweekly laboratory testing and was monitored for any signs and/or symptoms of liver failure. He was advised to continue UDCA, as well as to maintain a bland diet and adequate hydration. This led to rapid decline in total bilirubin levels from 28.9 to 4.0 mg/dL in next few weeks (Table 1). He was advised to avoid any use of kratom in future. UDCA was discontinued after 4 weeks following improvement in liver panel.

**Table 1.** Trend of Serum Liver Chemistries From Initial Presentation.

Days From Initial Presentation	Bilirubin (mg/dL)	AST (U/L)	ALT (U/L)	ALP (U/L)	GGT (U/L)	Ferritin (ng/mL)
Normal values	<1.4	<40	<60	40-130	<60	8-282
Day 1	22.8	48	62	259	N/A	N/A
Day 2	22.3	45	56	262	N/A	N/A
Day 3	23.5	48	57	287	N/A	1440.1
Day 11	28.9	41	34	301	N/A	N/A
Day 16	22.3	55	42	292	60	1279.4
Day 27	4.0	71	78	183	106	N/A

Abbreviations: AST, aspartate aminotransferase; ALT, alanine aminotransferase; ALP, alkaline phosphatase; GGT, γ-glutamyl transferase.

## Discussion

The main active alkaloid in kratom is mitragynine and 7-hydroxymitragynine, which acts mainly through monoaminergic and opioid receptors.^3,5^ It has multiple clinical properties including sedation-like effects similar to opioids. Several adverse effects can occur secondary to kratom ingestion, which include nausea, weight loss, insomnia, constipation, and increase in urinary frequency.^3,6^ There is also a growing risk of abuse with its chronic use given its opiate-like and stimulant-like properties along with failure to detect its metabolites in routine drug screening testing kits. Kratom use can also lead to significant drug interactions though the evidence is only limited to case reports/case series.^5^

We report a biopsy-proven acute cholestatic liver injury secondary to use of kratom. Majority of the patients with DILI are asymptomatic on presentation but can be noted to experience anorexia, abdominal pain, jaundice, malaise, and low-grade fever. Jaundice was the presenting sign in our patient that led to workup and eventual diagnosis of kratom-related DILI. UDCA is often used to treat various cholestatic liver diseases.^7^ It is a hydrophilic bile acid that acts via 3 proposed mechanisms of action: protecting cholangiocytes from hydrophobic and cytotoxic bile acids, stimulating the secretion of bile along with toxic substances, and preventing hepatocellular apoptosis as a result of accumulated bile acids.^7^ While UDCA tends to be used for its FDA-approved indication in cholestatic liver disease, primary biliary cholangitis, it has also been used in the setting of an experimental protocol in primary sclerosing cholangitis, intrahepatic cholestasis of pregnancy, liver disease in cystic fibrosis, progressive familial intrahepatic cholestasis, chronic graft-versus-host disease, and drug and parenteral nutrition-induced cholestasis.^7^ One hypothesis suggests that UDCA induces “a bile acid-, drug-, and cholesterol-metabolizing enzyme” known as cytochrome P450 3A4 (CYP3A4).^8^

Our patient improved with kratom abstinence along with UDCA use. Initially, bilirubin continued to rise despite being on UDCA. However, later the total bilirubin levels improved and downtrended. One possible explanation for this is that liver injury may continue after stopping the offending drug, which might be due to prolonged half-life of the drug. Kapp et al reported detecting metabolites of kratom in urine 2 weeks after abstinence.^9^ Even though UDCA was used to treat our patient, there have been a few other similar cases in which the conservative management alone with discontinuation of kratom resulted in improvement. For instance, Dorman et al reported a case of cholestatic hepatitis secondary to kratom consumption and revealed reversal when kratom was stopped and reoccurrence with a shorter latency period when restarted.^10^ There was another reported case of a patient who presented with jaundice and hyperbilirubinemia about 2 to 3 weeks after taking kratom twice a day for 4 days; after a negative workup for known etiologies of liver disease, and unrevealing abdominal imaging, he was diagnosed with herb-induced liver injury with cholestasis, which was successfully treated with supportive care alone.^11^ N-acetylcysteine has also shown to be effective in kratom-induced liver injury but again evidence is limited.^12^

Although the patient was on acetaminophen, he was only ingesting recommended doses. There is a possibility that acetaminophen might have enhanced the injurious effects of kratom but seems unlikely being only on 800 mg twice daily dose. Some may still argue that causality between kratom use and liver disease onset has yet to be confirmed.^13^ Kratom consists of more than 35 chemical products along with contaminants from extraction, and it is difficult to determine what exactly causes the liver injury.^10^ Furthermore, kratom has been reported to be toxic when taken at higher doses with prolonged consumption as opposed to lower, safer doses; along with other herbal supplements, it has also been reported to have harmful effects only in Western countries as opposed to being used in Southeast Asia for many years, implying that the effects could be from contamination, misidentification, or preparation errors.^10,13^ Well-designed studies are needed to answer these important questions and describe the therapeutic mechanism of kratom if any.

## Conclusion

Use of kratom is associated with acute intrahepatic cholestasis liver injury that can predispose to risk of progressive hepatic decompensation and liver failure. Therefore, caution is warranted, and use of kratom must be avoided until well-designed studies can be conducted. Enforcement of strict FDA regulations in the formulation and manufacturing of herbal supplements combined with improved public awareness and education regarding the current limitations in terms of lack of robust scientific studies before a herbal supplement can be marketed as a health product may promote a cautious approach by manufacturers and public to minimize the potential of adverse effects.
